# A Quick, Simple, and High-Throughput Method for Determining Li/Mg, Mg/Ca, Sr/Ca, and U/Ca Ratios in Coral Skeleton Using Quadrupole ICP-MS

**DOI:** 10.1155/jamc/6326611

**Published:** 2025-03-04

**Authors:** Klimentsi Cherviakouski

**Affiliations:** Analytical Core Lab, King Abdullah University of Science and Technology, Thuwal 23955, Saudi Arabia

## Abstract

Information about sea surface temperature (SST) allows both local and global climate reconstructions dating back centuries to millennia. To determine SST variations over time, including the deep past, a number of proxies are used. Li/Mg, Mg/Ca, Sr/Ca, and U/Ca ratios in coral skeletons are the most commonly used high-resolution temperature proxies. Various methods, using different types of instrumentation, are employed to obtain high-precision data on the variation of these ratios in coral skeletons. Generally, each method has its own advantages and disadvantages; however, a common drawback of most is that they are time-consuming. This article presents a quick, simple, and high-throughput method for determining mentioned ratios in coral skeletons using quadrupole ICP-MS. The reduction of calcium concentration to 3 ppm in the analytical solutions, combined with optimized operating conditions and quasi-simultaneous measurement of each element pair, ensures excellent signal sensitivity and stability of ratio values during sample runs. The drift of ratio values was investigated using the in-house coral secondary standard NEP-3b. During some runs, the drift of ratios during the analysis was so low that it provided excellent repeatability of results without off-line mathematical correction of the obtained data. Due to the selected ICP-MS operating conditions, this high-sensitivity method enables for the analysis of all studied isotopes in a single measurement, whereas typical procedures require two sample preparations and separate measurements. As a result, this method doubles the throughput compared to the previous procedures while demonstrating comparable short- and long-term precisions, both of which were assessed using the international standard sample JCp-1.

## 1. Introduction

The geochemical analysis of annual bands of coral skeletons provides the opportunity to extend the knowledge in paleoclimatology and climatology. The variation of ratio values Li/Mg, Mg/Ca, Sr/Ca, and U/Ca in annual coral bands reflects annually sea water surface temperature changes which can be traced in a long time period [1]. High-precision measurements of Li/Mg, Mg/Ca, Sr/Ca, and U/Ca are required to reconstruct past sea surface temperatures (SSTs). For example, Sr/Ca changes by only 0.06 mmol/mol, or 0.67%, with each degree Celsius [2]. The isotope dilution thermal ionization mass spectrometry (ID-TIMS) allows to perform measurements with an uncertainty of better than 0.1% [3, 4]. However, slow sample throughput and high cost restrict its extensive application. ICP-MS methods of the analysis are among the most common techniques replacing the ID-TIMS method. High-resolution sector field spectrometers (SF-ICP-MS) with multicollector detector systems (MC-ICP-MS) are considered the best alternatives to ID-TIMS instruments. However, in practice, significantly cheaper quadrupole (Q-ICP-MS) spectrometers are increasingly being used for the element-to-calcium ratio analysis in coral skeletons [5–16]. This shift is due to the rapid development and improvement of this type of mass spectrometer.

Typically, because the concentrations of trace elements (Li and U) in coral skeletons are 3-4 orders of magnitude lower than those of minor elements (Mg and Sr), two different analytical solutions are prepared and analyzed to measure all these elements in a single sample. Commonly, the Ca concentration in the solutions is 10 ppm for the Mg and Sr analysis, and 100 ppm for the Li and U analysis [8–10, 12, 14]. However, the deposition of Ca salts on instrument cones during measurements, along with mass discrimination and unstable room temperature, causes substantial drift in isotope ratio values during sample runs. To minimize errors induced by this drift, an obligatory off-line mathematical correction of all measurements is generally performed using a standard bracketing method. Usually, a drift correction standard (DCS) with a comparable matrix is measured after every three to 10 samples to monitor isotope signal drift during a sample run. A linear interpolation of the intensity variation between each pair of DCS measurements is then applied to each isotope [11]. This correction method improves both the accuracy and repeatability of the analysis. However, its effectiveness is influenced by the magnitude of the intensity variation between two DCS measurements. Correction for low-intensity variations yields better results compared to high-intensity variations, as linear interpolation may be inaccurate and, consequently, less effective for high-intensity changes. The intensity variation can be reduced by either increasing the number of DCS measurements during a run or by decreasing instrument drift. While the first option is straightforward to implement, it extends the analysis time and worsens the method's throughput. In the proposed method, drift in isotope ratio values during sample runs was reduced by decreasing the Ca concentration in the analyzed solutions to 3 ± 0.3 ppm and optimizing the analysis conditions for each isotope pair. The element ratio drifts were sometimes so low that mathematical corrections for the corresponding measurements could be omitted. Combined with the ability to measure all investigated ratios in a single analytical solution, the developed method ensures high throughput.

## 2. Materials and Methods

### 2.1. Isotope Selection

The next isotopes ^7^Li, ^24^Mg, ^88^Sr, ^238^U, and ^48^Ca were selected for monitoring in this study.  Table 1 presents the primary interferences affecting the analyzed isotopes and the techniques employed to minimize them. Additional potential interferences for ^48^Ca from ^32^S^16^O and for ^24^Mg from ^12^C_2_ are considered negligible. This is due to the low sulfur concentration (less than 0.5% [17, 18]) in coral skeletons and the low carbon content in the analyzed solutions, as carbon is removed during sample preparation as CO_2_. Regarding isobaric interference ^48^Ti on ^48^Ca, it is also negligible, due to the trace levels of titanium in coral skeletons [19].

### 2.2. ICP-MS: Analyzed Modes and Operation Conditions

All measurements of each ratio were conducted on the triple quadrupole ICP-MS Agilent 8800, equipped with a microflow nebulizer and standard Ni-Cu sample and skimmer cones. The MassHunter Workstation Software version 4.4 enabled individual element pair measurements in No Gas mode during a sample analysis. A short dwell time of 100 µs for both pulse-counting and analog modes of the electron multiplier detector allowed for quasi-simultaneous measurements of elements in both modes. Isotopes of ^7^Li and ^238^U were registered in the pulse-counting regime, while ^24^Mg, ^88^Sr, and ^48^Ca were measured in the analog regime. To increase the accuracy and precision of Sr/Ca ratio measurements, the Sr-Ca pair was analyzed twice in identical modes. Six calibration curves were employed in the analysis: two single-element curves (Li and Mg) and four ratio curves (Mg/Ca, two for Sr/Ca, and U/Ca). The correlation coefficients for the single-element calibration curves were not less than 0.9998, while the correlation coefficients for the ratio calibration curves were not less than 0.9999. The total time for one sample analysis, including instrument washing, was 255 ± 2 s. The main operational conditions in all five modes are outlined in Table 2.

The presented plasma parameters were selected primarily to maximize the intensity of the Li isotope, while the acquisition parameters minimized signal fluctuations for all isotopes.

### 2.3. Reagents, Standards, and Reference Materials

High purity deionized water (18.2 MV cm) was obtained from water purification systems Milli-Q purification system (Millipore, Bedford, MA, USA). Nitric acid (ACS, 70% nitric acid, Sigma-Aldrich, Germany) was further purified by a sub-boiling distillation system (duoPUR model, Milestone, Italy) at power 10 W before exploitation. All working standard solutions were prepared by appropriate dilution of monoelemental certified stock solutions (Inorganic ventures, United States): MSLI-10PPM (Li, 10 mg/L), MSU-10PPM (U, 10 mg/L), CGMG1 (Mg, 1000 mg/L), CGSR1 (Sr, 1000 mg/L), and CGCA1 (Ca, 1000 mg/L). The international coral standard JCp-1and in-house coral secondary standard NEP-3b were used to assess the accuracy and reproducibility of the results.

### 2.4. Sample and Reference Materials' Dissolution

All samples and reference materials were dissolved and diluted in such manner to receive Ca concentration in final analytical solution 3 ± 0.3 ppm. This is the minimum Ca concentration at which the trace element Li, after dilution, still produced a signal suitable for the analysis. Analytical portion 15.0 ± 1.5 mg of the coral powder sample was weighted and placed into 15 mL centrifuge tube and completely dissolved with 2.0 mL of 4% v/v nitric acid solution followed by the addition of 6.0 mL of DI water. Because of the limited quantity of reference materials available for the analysis, only about 2.5 mg each of JCp-1 and NEP-3b was used for analytical solutions' preparation. Due to the substantial reduction in the mass of the reference samples, the volumes of the 4% v/v nitric acid solution and deionized (DI) water used for sample dissolution were decreased to 0.3 mL and 0.9 mL, respectively. Both sample and reference material solutions were centrifuged at 4000 rpm for 10 min. To obtain a final analytical solution with a calcium concentration of 3 ± 0.3 ppm, aliquots of 38 ± 2 μL from the reference material supernatant and 50 ± 10 μL from the sample supernatant were diluted in 10.0 mL of 1% v/v nitric acid solution. The dilution factor for both CRMs and samples depended on the mass taken for the analysis and was approximately 3.3 × 10^5^. After equilibrating for at least 2 h, the diluted solutions were analyzed.

### 2.5. Calibration Standards' Preparation

Using monoelemental certified stock solutions, the intermedia standard solution with concentration of Li 1.25 ppb, Mg 2.0 ppm, Sr 12.5 ppm, U 7.5 ppb, and Ca 3 ppm in 1% v/v nitric solution was prepared. It has been stored in an FEP bottle during the period of all experiments and used for the calibration standard preparation. The calibration standards were prepared in 50 mL falcon tubes by the dilution intermedia standard solution in the next proportions 1:500, 2:500, and 3:500 with 1% v/v nitric acid solution containing 3 ppm of Ca. The obtained concentrations of all elements in the calibration solution are shown in Table 3.

The calibration standards have been used and stored for a short time period, specifically less than 10 days.

### 2.6. Calibration Curves' Creation

**Figure 1:**
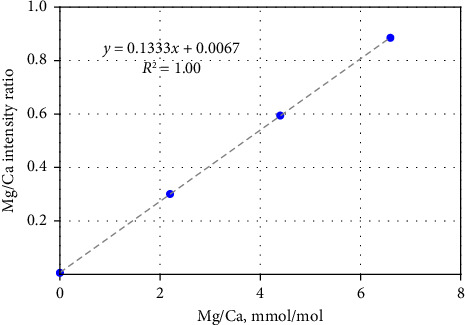
The example of the calibration curve for the Mg/Ca ratio analysis.

To minimize postanalysis calculations for element-to-calcium ratios, in MassHunter Workstation Software, calcium was analyzed as an internal standard. The calibration curves of ^24^Mg/^48^Ca, ^88^Sr/^48^Ca, and ^238^U/^48^Ca ratios were created based on concentration of these elements in calibration standards. These ratios were expressed in mmol/mol units for the first two and μmol/mol for the last. The example of the calibration curve for the Mg/Ca ratio analysis is presented in Figure 1. Calibration curves for the individual isotope ^24^Mg were created based on its concentration in standard solutions at 4, 8, and 12 ppb (Table 3). For ^7^Li, concentrations of 2.5, 5.0, and 7.5 ppt (Table 3) were multiplied by a coefficient of 3.5017, which corresponds to the ratio of the molar masses of ^24^Mg and ^7^Li. Due to described manipulations, the software provided the values of Mg/Ca, Sr/Ca, and U/Ca ratios directly after each sample analysis. For the Li–Mg pair, only one additional mathematical operation was required: dividing the Li results by the Mg results to obtain the Li/Mg ratio in mmol/mol units.

### 2.7. Instrument Tuning and Sample Analysis

Before starting the instrument tuning, the instrument was warmed up for at least 1 hour. This was followed by preconditioning the nickel cones for a minimum of 30 min with a sample solution containing 3 ± 0.3 ppm Ca in 1% HNO_3_. Since three modes have identical operating conditions (Table 2), tuning was performed for Modes 1, 3, and 5 using a standard tune solution (1 ppb of Ce, Co, Li, Tl, and Y). To reduce the Li memory effect, the tune solution was diluted by a factor of 5. Mode 1 and Mode 3 were tuned using isotopes ^7^Li and ^59^Co with mass shifts of 0% and 20%, respectively. Mode 5 was tuned with isotopes ^59^Co and ^205^ Tl at a mass shift of −100%. After completing the tuning, the obtained operating ICP-MS parameters for Mode 3 were transferred to Modes 2 and 4. Since the selected isotopes of the investigated elements were either free from polyatomic and doubly charged ion interferences, or the impact of these interferences was minimized (as shown in Table 1), the ICP-MS operation conditions were optimized primarily to achieve maximum intensity for Li and U isotopes without prioritizing interference minimization. Under these selected conditions, the CeO+/Ce+ and Ce2+/Ce + values were slightly higher than the typical 2%, ranging from 4% to 6% and 6% to 9%, respectively.

Using the described analytical procedure, approximately 400 coral skeleton samples were analyzed over a period of about 10 months. During this time, four batches of samples were delivered to the laboratory. Two reference materials, the international coral standard JCp-1 and the in-house coral secondary standard NEP-3b, were dissolved along with the samples from each batch. The secondary standard NEP-3b was used to monitor signal drifts of the studied isotopes and apply mathematical corrections when necessary. This standard was typically analyzed seven times in each run. The international coral standard JCp-1 was analyzed at the beginning, middle, and end of each run, totaling 4-5 measurements. On average, 33 unknown samples were analyzed daily over a period of approximately 3.5 h.

## 3. Results and Discussion

### 3.1. Background

In the current study, highly diluted sample solutions were utilized for the analysis. As a result, the concentrations of the elements of interest, particularly lithium and uranium, were very low (Table 4). In this case, it was crucial to either keep the background-to-sample signal ratio close to zero or minimize the difference in this ratio between the blank and analyzed samples. According to Table 4, for isotopes ^88^Sr and ^238^U, the background-to-sample signal ratio was < 0.0005, and, as a result, it could not influence the analysis results. However, this signal ratio for ^7^Li and ^24^Mg isotopes was 0.028 and 0.009, respectively, and could not be ignored. For the ion ^7^Li+, two potential sources of the background signal were identified: lithium contamination in the standard solution CGCA1 (Ca, 1000 mg/L), which was used for calibration standard preparation, and double-charged ^14^N++ ions. Lithium contamination in the calcium standard solution could introduce a systematic error in the analysis. This would affect accuracy by lowering the results, but it would not impact precision. Since all solutions were prepared with the same calcium content of 3 mg/L throughout the analysis, slight deviations from this concentration during the preparation would not have a noticeable effect on precision. Using solutions with the same concentration of HNO_3_ (1%) for both calibration standards and analyzed samples compensated for the double-charged ion interference from ^14^N++. Regarding the background-to-sample signal ratio for ^24^Mg+, the main source of the background signal, namely, ^48^Ca++ double-charged ions, was compensated by maintaining a similar Ca content in both calibration standards (3 ppm) and samples (3 ± 0.3 ppm).

### 3.2. Ratios' Drift and Short-Term Repeatability

**Figure 2:**
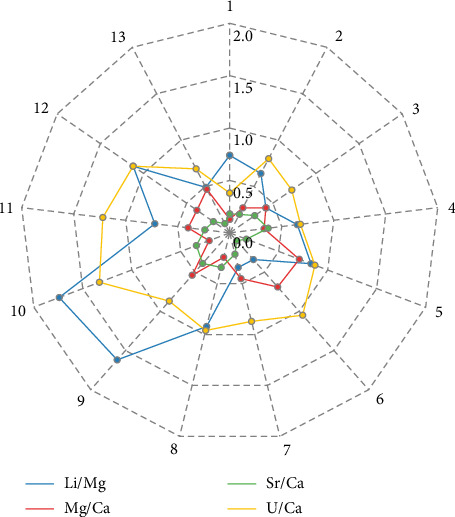
The RSD of the investigated ratio values, obtained from the NEP-3b analysis within each run of samples.

Mass discrimination (or mass bias) occurs for all kinds of ICP systems. This phenomenon can be defined as the deviation of measured isotope ratios from the “true value” due to the different transmission of ions according to their masses before the final detection [20]. In our case, mass discrimination was corrected using external matrix-matched calibrations. However, modern ICP-MS systems often experience slow drift during extended analytical runs. This drift, typically manifesting as a decrease in signal intensities, is mainly attributed to unstable room temperature, varying plasma conditions, and changes in the interface and ion lens systems. To monitor the drift of the studied isotope ratios and apply mathematical corrections, when necessary, the NEP-3b standard sample was measured typically every seven samples throughout the batch. If the within-batch relative standard deviation (1σ, % RSD) of the Li/Mg, Mg/Ca, Sr/Ca, and U/Ca ratios measured in NEP-3b was higher than 0.9%, 0.5%, 0.3%, and 0.5% respectively, an off-line correction of data was performed by a standard bracketing method. The critical % RSD values chosen are based on the results reported by Hathorne et al. [1] and Ross et al. [10]. The obtained RSD values for each interested ratio measured in NEP-3b over 13 runs are shown in Figure 2.

**Figure 3:**
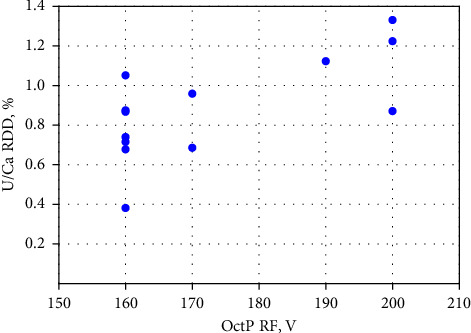
Dependence of the RSD of the U/Ca ratio on the value of OctP RF.

According to the results presented in Figure 2, the RSD values of most ratios (29 out of 52) were within acceptable limits and did not require mathematical correction. However, the nearly constant drift observed in 12 out of 13 cases, for the ^238^U/^48^Ca ratio, necessitated correction. This can be attributed to the fact that the individual signal fluctuations and drifts of these isotopes signals were not synchronized, due to the significant mass difference between them.  Figure 3 demonstrates that the repeatability values of the ^238^U/^48^Ca ratio depend on the OctP RF value. Higher RSD magnitudes were observed at the maximum of 200 V or near-maximum of 190 V for OctP RF. These high values of OctP RF caused significant signal drift of low-mass calcium isotope. Since after autotune of Mode 5 with a 100% mass shift, the OctP RF value is often automatically set to 190 or 200 V, and we recommend manually adjusting the OctP RF to 160 V in such cases.

**Figure 4:**
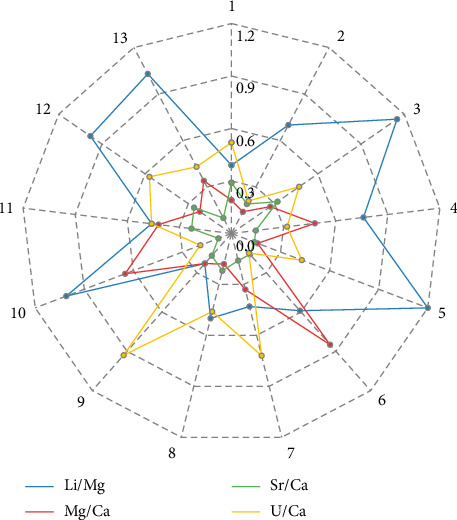
The RSD of the investigated ratio values, obtained from the JCp-1 analysis and correction within each run of samples.

Figure 2 also shows that, for other isotope ratios, the ratio signals generally maintained good stability during a single run. The calculated RSD values were close to or below permissible limits. The repeatability of results (short-term precision) for each ratio was estimated based on measurements of the standard sample JCp-1 during each of the 13 runs. The calculated, and if necessary, corrected RSD values for all ratios measured in JCp-1 are presented in Figure 4. The short-term precision, calculated as the average RSD of these values, was as follows: 0.82% for Li/Mg, 0.40% for Mg/Ca, 0.20% for Sr/Ca, and 0.49% for U/Ca.

The results presented above demonstrate that the current method ensures relatively low drift for all ratios, which can be successfully corrected.

### 3.3. Long-Term Precision and Accuracy

The long-term precision (reproducibility) of the proposed procedure, expressed as the relative standard deviation (1σ RSD, %), was determined from the ratio analysis in standard samples JCp-1 over a period of approximately 10 months. Accuracy was evaluated by comparing the ratio values, calculated as the average from 58 measurements of JCp-1, with those reported in other studies. The magnitudes described above are presented in Table 5.

According to Table 5, the proposed method ensures long-term precision for the studied ratios that are either better than or comparable to those reported in the previous studies [7, 12–14, 21, 22] and in the international laboratory comparison [1], which involved 21 different laboratories. Regarding the accuracy of the current method, the obtained ratio values demonstrate a good correlation with results from the international laboratory comparison [1], which are now considered as reference values. A comparison with values reported earlier in other investigations confirms the suitable accuracy of the developed method.

## 4. Conclusion

The quick, simple, and high-throughput method for determining Li/Mg, Mg/Ca, Sr/Ca, and U/Ca ratios in coral skeletons using quadrupole ICP-MS has been developed in the current work. By reducing the calcium concentration to 3 ± 0.3 ppm in the analyzed solution and optimizing the analysis parameters, the method enabled the measurement of all investigated isotopes from a single sample, significantly increasing throughput. Moreover, the high stability of the ratio values during the sample run allowed for the omission of off-line mathematical correction in most cases, simplifying postanalysis mathematical treatments. At the same time, the proposed method has demonstrated short- and long-term precision and accuracy better or comparable to the previous methods. These features make it one of the preferred methods for the routine analysis of element-calcium ratios in coral skeletons within the context of climatological studies.

## Figures and Tables

**Table 1:** The main interferences on the selected isotopes and techniques of minimization.

Analyte ion	Interfering ion	The techniques of minimization
^7^Li+	^14^N++	Same concentration of HNO_3_ 1% in calibration standards and samples
^24^ Mg+	^48^Ca++	Similar content of Ca in calibration standards 3 ppm and samples 3 ± 0.3 ppm
^88^Sr+	^48^Ca^40^Ar	Similar content of Ca in calibration standards 3 ppm and samples 3 ± 0.3 ppm
^48^Ca+	^14^N^18^O^16^O	Same concentration of HNO_3_ 1% in calibration standards and samples

**Table 2:** Main operation conditions.

		Mode 1	Mode 2	Mode 3	Mode 4	Mode 5
Isotope		^7^Li, ^24^Mg	^24^Mg, ^48^Ca	^88^Sr, ^48^Ca	^88^Sr, ^48^Ca	^238^U, ^48^Ca

*Acquisition parameters*						
Peak pattern		3				
Replicates		3				
Sweeps		400				
Integrate time	s	6.0; 0.4	0.4; 0.4			4.0; 0.4

*Plasma*						
RF power	W	1300				
Sample depth	mm	6.5				
Nebulizer gas⁣^∗^	mL/min	0.98				
Nebulizer pump	rmp	0.15				
Spray chamber temp	°C	2				
Makeup gas⁣^∗^	mL/min	0.13				

*Lenses*⁣^∗∗^						
Extract 1	V	−67.9	−67.9			−68.0
Extract 2	V	−200.0	−200.0			−200.0
Omega bias	V	−120	−115			−120
Omega lens	V	10.0	9.3			6.8

*Cell*						
OctP bias	V	−14	−25			−25
OctP RF⁣^∗∗^	V	120	130			160

⁣^∗^Manually could be slightly changed to decrease ratios: CeO^+^/Ce^+^ and Ce^2+^/Ce^+^.

⁣^∗∗^Automatically could be changed during autotune.

**Table 3:** Concentrations of investigated elements in calibration standard solutions, μg/L.

	Li	Mg	Sr	U	Ca
Blank	0.000	0.0	0.0	0.000	3000
Cal. STD 1	0.0025	4.0	25.0	0.015	3000
Cal. STD 2	0.0050	8.0	50.0	0.030	3000
Cal. STD 3	0.0075	12.0	75.0	0.045	3000

**Table 4:** The typical intensity of isotopes' signals in blank solutions and sample solutions at normal concentration of elements in the coral skeleton.

		Li	Mg	Sr	U	Ca
Blank	Conc, μg/L	0.000	0.000	0.000	0.000	3000
	Intensity, counts	65	3.7·10^4^	2.0·10^4^	3.5	6.0·10^6^

Samples	Conc, μg/L	0.003	7.5	60	0.020	3000
	Intensity, counts	2.3·10^3^	4.0·10^6^	4.1·10^7^	1.0·10^4^	6.0·10^6^

**Table 5:** The average values and relative standard deviations of the ratios in the standard JCp-1.

Elemental ratio	References	Average, mmol/mol	1*σ*, RSD, %	*n*
Li/Mg	This study	1.54	0.94	58
	D'Olivo et al. [12]	1.47	0.52	17
	Fowell et al. [21]	1.51	1.99	16
	Montagna et al. [7]	1.43	3.5	
	Hathorne et al. [1]	1.49	2.9	4⁣^∗^
	Hathorne et al. [22]	1.51	2.6	25
	Thomson et al. [14]		1.83	19

Mg/Ca	This study	4.25	0.66	58
	D'Olivo et al. [12]	4.20	0.45	17
	Montagna et al. [7]	4.21	1.19	
	Hathorne et al. [1]	4.20	1.55	19⁣^∗^
	Hathorne et al. [22]	4.17	1.35	25
	Thomson et al. [14]		0.42	19

Sr/Ca	This study	8.86	0.29	58
	D'Olivo et al. [12]	8.85	0.21	17
	Fowell et al. [21]	8.66	1.15	20
	Hathorne et al. [1]	8.84	0.48	21⁣^∗^
	Thomson et al. [14]		0.32	19
	Cole et al. [13]	8.86	0.24	

U/Ca	This study	1.23·10^−3^	0.63	58
	D'Olivo et al. [12]	1.20·10^−3^	0.58	17
	Hathorne et al. [1]	1.19·10^−3^	3.78	8⁣^∗^
	Hathorne et al. [22]	1.18·10^−3^	1.00	25
	Thomson et al. [14]		0.67	19

⁣^∗^Number of laboratories.

## Data Availability

The data used to support the findings of this study are included within the article.
